# Untangling the clonal architecture in a case of acute myeloid leukaemia with multiple cytogenetically unrelated clones

**DOI:** 10.46989/001c.124931

**Published:** 2024-11-12

**Authors:** Helen Cashman, Andrew J Wilson, Ke Xu, Elisabeth Nacheva, Robert Baker, Rajeev Gupta

**Affiliations:** 1 Department of Haematology University College London Hospitals NHS Foundation Trust; 2 Oncogenomics Department University College London Hospitals NHS Foundation Trust; 3 Department of Molecular Pathology/Genetics, Health Services Laboratory University College London Hospitals NHS Foundation Trust

**Keywords:** acute myeloid leukaemia, tetraploidy, MECOM, unrelated clones

Acute myeloid leukemia (AML) arises through the stepwise accumulation of transforming mutations in hematopoietic stem and progenitor cells. Leukemic cells exist in cellular hierarchies analogous to those seen in normal hematopoiesis, with the net result that in any AML, single cell analysis identifies multiple related leukemic sub-clones, harboring overlapping combinations of genetic mutations that define the bulk leukemia.^1^ In the diagnostic laboratory, cytogenetic and molecular analysis of bulk bone marrow provides all the information required for rapid risk assessment and treatment of new cases of AML. The hetero-cellular nature of AML is rarely apparent in this setting - although it may become more obvious through treatment as dominant clones emerge. Here we report a highly unusual exception to this in the case of a 34-year-old female presenting with AML during pregnancy. We were able to elucidate the likely clonal architecture of her AML by monitoring her clinical progress using routine diagnostic assays.

Our patient was a 36-year-old female who presented to hospital at 33 weeks pregnant with symptomatic COVID-19 infection. Blood tests on admission demonstrated pancytopenia: hemoglobin 89g/L [reference range (RR) 115 – 155g/L], white cell count (WCC) 1.28 x10^9^/L [RR 3 – 10x10^9^/L], neutrophils 0.47 x10^9^/L [RR 2 – 7.5x10^9^/L], platelet count 95 x10^9^/L [RR 150 – 400x10^9^/L] with 5% blasts on peripheral blood film. There was no significant past medical history apart from a current uncomplicated pregnancy. Bone marrow examination with flow cytometry confirmed acute myeloid leukemia with 82% blasts without specific phenotypic features on the aspirate smear (Figure 1a) and heavy infiltration of the trephine with blasts (80-90%). Targeted fluorescence *in-situ* hybridization (FISH) analysis detected both a tetraploid clone and a separate clone with rearrangement of the *MECOM* gene. The *MECOM* probe showed two separate populations, one diploid population with a *MECOM* rearrangement present at approximately 6% and a separate tetraploid population with four copies of intact *MECOM* at approximately 11-15% (Figure 1b). None of the tetrasomic clones demonstrated a *MECOM* rearrangement. Chromosomal microarray analysis (CMA) (8x60K oligonucleotide arrays, Agilent) did not detect any clinically significant imbalances (detection limit 20%). G banding analysis failed due to low cell growth and poor chromosome morphology. Two truncation mutations were detected by myeloid next generation sequencing (NGS) (Archer Variantplex) in *RUNX1* (Variant allele frequency [VAFs] 27% and 21%), and a truncation in *BCOR* (VAF 27%). RNASeq using the Archer Pan-Heme FusionPlex NGS assay demonstrated increased expression of multiple genes, including segments of *ETV6* and *JAK2* with a small volume *JAK2::ETV6* fusion. RNA was over-expressed in the whole of *BLNK, CDK6, CRLF2, DNTT, IRF8, STRBP, TCF3* and *TFG* (Table 1).

**Figure 1.**
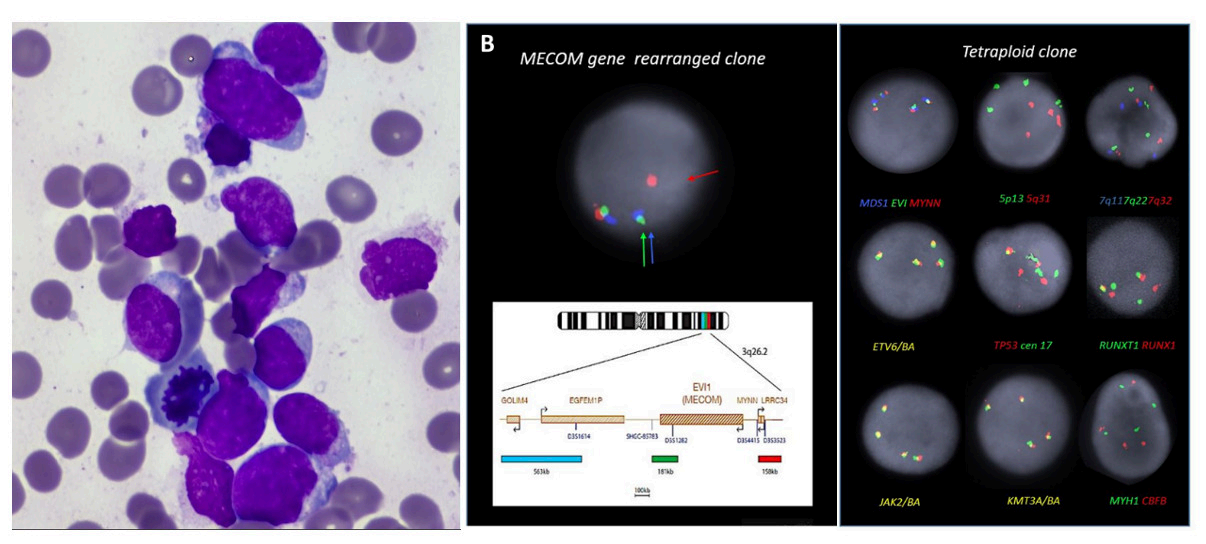
Bone marrow investigations at diagnosis^2^ 1a) Left panel, bone marrow aspirate morphology at diagnosis, demonstrating 82% blasts without specific phenotypic features; 1b) FISH at diagnosis demonstrating two separate clones; a tetraploid clone and a *MECOM* rearranged clone

**Table 1.** Investigation results at diagnosis and during treatment

**Time point**	**Morphological blast percentage**	**Flow cytometry**	**FISH/CMA**	**Molecular findings**
*Diagnosis*	BMA: 82% BMT: 80-90%	48% blasts Positive for CD34, CD117 (weak), HLA-DR, CD15, CD38, cCD34, cMPO and cTDT Negative for cCD79 and cCD3	FISH: -one diploid population with a *MECOM* rearrangement present -separate tetraploid population with four copies of intact *MECOM* CMA: female genome without any clinically significant imbalances	*BCOR* p.Arg1163Ter (VAF 27%), *RUNX1* p.Leu98SerfsTer24 (VAF 27%) and *RUNX1* p.Ala142LeufsTer3 (VAF 21%) *JAK2::ETV6* fusion Overexpression of *BLNK, CDK6, CRLF2, DNTT, IRF8, STRBP, TCF3* and *TFG*, 4 copies of *TP53*
*Post- FLA-IDA induction*	BMA 11% BMT: 5-10%	6.4% blasts Positive for CD34, CD117wk, CD33, CD13, HLA-DR, CD38	NAD	No mutations detected on BMA
*Post cycle 2 FLA-IDA*	BMA: 4% BMT: <5%	1% blasts	NAD	NT
*Shoulder joint aspirate prior to alloSCT*	Blasts not seen (cell clumping limiting morphological analysis)	Not performed	*MECOM* -rearranged clone 6% [23/380 cells]	NAD
*Post alloSCT*	BMA: 1% BMT: NT	<1% blasts	100% donor chimerism	NT

BMA – bone marrow aspirate, BMT – bone marrow trephine, NAD – no abnormalities detected, NT – not tested

Lower segment Caesarean section was performed at 34+5/40 weeks and induction therapy with FLA-IDA chemotherapy [fludarabine, cytarabine, idarubicin] was commenced six days following delivery. Repeat bone marrow examination after induction on day 20 demonstrated 11% blasts on aspirate smears and 6% by flow cytometry; however, the absence of abnormalities on FISH and molecular testing suggested regenerative blasts (Table 1). Complete morphological and cytogenetic remission was confirmed following the second cycle of FLA-IDA.

The patient developed a mycoplasma joint infection with a shoulder joint fluid aspirate revealing no morphological blasts; however, FISH confirmed a *MECOM* rearrangement in 6% of cells (23/380 cells) with no NGS abnormalities, suggesting extramedullary disease. The patient proceeded to matched unrelated donor allogeneic stem cell transplant (alloSCT) following bridging therapy with venetoclax/azacitidine. Post-transplant BMAT confirmed morphological, flow cytometric and cytogenetic remission with 100% male donor chimerism by FISH. NGS was not performed. At last follow up at Day +223 post alloSCT the patient remained in remission.

We report a case of AML with at least two seemingly cytogenetically unrelated leukemic clones including a tetraploid clone and a separate *MECOM*-rearranged clone in a pregnant patient. In addition, molecular analysis showed the presence of multiple somatic mutations: two *RUNX1* mutations and a *BCOR* mutation. At diagnosis, it was not possible to determine which abnormalities, if any, detected on molecular testing were associated with the two separate clones detected by FISH. During the treatment course, we utilized further sensitive testing to identify the *MECOM* rearranged blast population as the suspected dominant clone, due to persistent detection in joint fluid with clearance of other disease. Furthermore, the presence of the *MECOM* rearrangement in the shoulder joint aspirate without any concurrent detectable NGS abnormalities may suggest that the initial tetraploid clone was associated with the *RUNX1* and *BCOR* mutations.

Many acquired somatic mutations have been identified that can develop throughout the natural history of AML, including early pre-leukemic founder mutations insufficient for leukemia development in isolation and, later, secondary mutational events during linear evolution, culminating in overt leukemogenesis.^3–5^ Different leukemic clones can also develop during the treatment course resulting from loss or acquisition of mutations through branching evolution, and cytogenetic subclones are common.^3,6^ The detection of multiple cytogenetically unrelated co-dominant leukemic clones at diagnosis, as seen in our case, is rare, due to the inherent survival advantage usually gained by the dominant clone of leukemic hematopoietic stem cells at an early stage in development.^6,7^ Limited case reports and historical series have described the occurrence of cytogenetically unrelated leukemic clones in AML, which appears more common in MDS than AML.^7–10^ The presence of a *MECOM* rearrangement, *BCOR* and *RUNX1* mutations, and tetraploidy in AML are all independently associated with a poor prognosis.^10,11^ Despite the adverse risk profile, our patient demonstrated a favorable response to treatment, with complete remission achieved post-alloSCT. Utilizing sensitive testing methods, we were able to delineate the likely clonal architecture of the leukemic clones during her treatment journey and to demonstrate effective clearance of high-risk disease with chemotherapy and alloSCT.

## Consent to publish

The participant consented to submitting the case report to the journal.

## Statements and declarations

AW: Research funding – Gilead; Speakers fees – Gilead, Novartis, Jazz, Astellas.

HC, KX, EN, RB and RG: No conflicts of interest to disclose.
